# Environmental drivers of autumn migration departure decisions in midcontinental mallards

**DOI:** 10.1186/s40462-021-00299-x

**Published:** 2022-01-05

**Authors:** Florian G. Weller, William S. Beatty, Elisabeth B. Webb, Dylan C. Kesler, David G. Krementz, Kwasi Asante, Luke W. Naylor

**Affiliations:** 1 Missouri Cooperative Fish and Wildlife Research Unit, School of Natural Resources, University of Missouri, Columbia, MO 65211 USA; 2 U.S. Geological Survey, Upper Midwest Environmental Sciences Center, La Crosse, WI 54601 USA; 3 U.S. Geological Survey, Missouri Cooperative Fish and Wildlife Research Unit, Columbia, MO 65211 USA; 4 The Institute for Bird Populations, PO Box 1346, Point Reyes Station, CA 94956 USA; 5 Arkansas Cooperative Fish and Wildlife Research Unit, Department of Biological Sciences, University of Arkansas, Fayetteville, AR 72701 USA; 6 Environmental Systems Research Institute (Esri), 3325 Springbank Ln # 200, Charlotte, NC 28226 USA; 7 Arkansas Game and Fish Commission, Little Rock, AR 72205 USA

**Keywords:** Autumn migration, Mallard, Satellite tracking, Discrete choice model, Mississippi Flyway

## Abstract

**Background:**

The timing of autumn migration in ducks is influenced by a range of environmental conditions that may elicit individual experiences and responses from individual birds, yet most studies have investigated relationships at the population level. We used data from individual satellite-tracked mallards (*Anas platyrhynchos*) to model the timing and environmental drivers of autumn migration movements at a continental scale.

**Methods:**

We combined two sets of location records (2004–2007 and 2010–2011) from satellite-tracked mallards during autumn migration in the Mississippi Flyway, and identified records that indicated the start of long-range (≥ 30 km) southward movements during the migration period. We modeled selection of departure date by individual mallards using a discrete choice model accounting for heterogeneity in individual preferences. We developed candidate models to predict the departure date, conditional on daily mean environmental covariates (i.e. temperature, snow and ice cover, wind conditions, precipitation, cloud cover, and pressure) at a 32 × 32 km resolution. We ranked model performance with the Bayesian Information Criterion.

**Results:**

Departure was best predicted (60% accuracy) by a “winter conditions” model containing temperature, and depth and duration of snow cover. Models conditional on wind speed, precipitation, pressure variation, and cloud cover received lower support. Number of days of snow cover, recently experienced snow cover (snow days) and current snow cover had the strongest positive effect on departure likelihood, followed by number of experienced days of freezing temperature (frost days) and current low temperature. Distributions of dominant drivers and of correct vs incorrect prediction along the movement tracks indicate that these responses applied throughout the latitudinal range of migration. Among recorded departures, most were driven by snow days (65%) followed by current temperature (30%).

**Conclusions:**

Our results indicate that among the tested environmental parameters, the dominant environmental driver of departure decision in autumn-migrating mallards was the onset of snow conditions, and secondarily the onset of temperatures close to, or below, the freezing point. Mallards are likely to relocate southwards quickly when faced with snowy conditions, and could use declining temperatures as a more graduated early cue for departure. Our findings provide further insights into the functional response of mallards to weather factors during the migration period that ultimately determine seasonal distributions.

**Supplementary Information:**

The online version contains supplementary material available at 10.1186/s40462-021-00299-x.

## Background

Avian seasonal migration is an energetically costly series of movements that may cover great distances, and is fundamental to the ecology of many bird species [1, 2]. Migration decisions can influence fitness in both the breeding and non-breeding portion of the year. The timing, distance and speed of migratory movements can influence and be influenced by body condition, reproductive success, and population composition [3, 4] and has been shown to influence long-term processes including continental or global distribution and speciation [5].

Understanding migratory movements in waterfowl has been considered particularly important, due to their status as popular gamebirds with associated population and habitat management programs [2, 6] as well as their role in the spread of zoonotic diseases [7]. Many waterfowl, and most ducks, depend on wetlands that are particularly threatened by anthropogenic climate and land use change [8, 9]. Phenological shifts in waterfowl migration have been connected to altered habitat conditions and weather patterns in both breeding and wintering ranges [9–11].

Although an increasing number of studies have recently been published about the proximal factors influencing migration chronology in waterfowl [12–18], much remains unclear. There is a lack of quantitative knowledge about the parameters that drive migration timing, distance, and choice of target location among waterfowl [2, 19], with the information scarcity more pronounced for autumn than spring migration [18]. Response to decreasing photoperiod is thought to be the principal external cue for autumn departure in many bird species, especially song birds [2, 20, 21], but this has not yet been clearly demonstrated in waterfowl. Among northern hemisphere waterfowl, decision to embark on southwards migration is thought to principally depend on energy budget considerations. With declining food availability under autumn and winter conditions, metabolic costs increase and food becomes harder to acquire, until staying at high latitudes is more costly than expending energy to relocate southward [12, 22, 23]. The effect of decreasing temperatures together with the occurrence of snow and ice cover are principal migration cues in ducks and geese, both in the short term and as cumulative measures over longer periods [2, 6, 12, 15, 18].

On a proximate time scale, decision to migrate is frequently influenced by how energetically favorable weather conditions are on a given day for long-distance movements [1]. Flight may be made costly by headwinds or facilitated by tailwinds [17, 24], precipitation may impede flight and increase thermoregulation costs [1, 17, 25], cloud cover may obstruct the view of visual cues used for navigation [26], and pressure differences may serve as cues for impending weather changes [1] or facilitate departure [27]. Flight weather is a prominent migration driver in passerines [19, 25, 28], but with the notable exception of wind direction [17, 18, 29, 30], it plays less of a role in waterfowl [31, 32]. However the majority of existing studies focused on shifts in waterfowl abundance at the population level, and were generally restricted to few chosen locations at particular latitudes. There is still much less information on what drives movement decisions at the individual level, and across the latitudinal range between breeding and wintering grounds. However location records from satellite-tracked birds are increasingly used to address this information gap [13, 14, 16, 18, 33].

The mallard (*Anas platyrhynchos*) is the most numerous species among ducks migrating through the Mississippi Flyway and considered a priority in the development of wetland management plans and hunting regulations [34, 35], with an average of 2.8 million individuals each year wintering in the Mississippi Alluvial Valley alone [36]. Consequently, the timing and drivers of mallards’ migratory movements into and through the flyway are of great interest for the development of accurate population models for conservation planning in the region [16, 37, 38]. In this study we investigated the autumn migration movements of satellite-tagged mallards within the Mississippi Flyway. Our objective was to evaluate individual-level decisions to embark on migration movements as a function of environmental drivers. As the migration process depends on bird behaviour over a sequence of directional movements, and extended stays at stopover sites are common for many waterfowl and especially ducks [2, 39], we were interested to study departure and stopover events both inside and outside the wintering range. We used a discrete choice modeling framework [40] to develop resource selection functions [41], while including environmental covariates that could account for the impact of winter conditions on local habitat and for the short-term energetic efficiency of undertaking migration flights. Based on the findings reported in the literature, we hypothesized that mallard’s decisions to relocate would be principally driven by the onset of longer-term winter conditions (snow and falling temperatures) and modified by short-term conditions that may facilitate or impede embarking on a long-distance flight (wind, precipitation, and visibility), and that these relationships would be applicable across the migration range.

## Methods

### Location data

We used two existing sets of location records from satellite-tracked mallards with a combined size of 220 individuals, of which 43 were eventually used for analysis. Data set A consisted of 180 mallards of both sexes that were captured and tagged in several locations in Arkansas in February–March 2004 (23 female: 10 male), February 2005 (27 female: 21 male), January–February and November–December 2006 (39 female: 15 male), and January–February 2007 [45 female]. Transmitter units weighed 22–35 g (1.8–2.6% of body mass at capture [mean ± S.D.: 1098 ± 132 g]) and used the CLS-Argos (Toulouse, France) satellite system to monitor movement [42]. For details on tagging and transmitters see [14].

Data set B was gathered from 40 mallard hens, of which 20 were captured in the same location in Saskatchewan in September 2010, and 20 in February 2011 in multiple location in Arkansas. Transmitter units weighed 28 g (2.4–2.7% of body mass at capture [mean ± S.D.: 1099 ± 71.5 g]) and used the GPS satellite system to monitor movement. We used a version of the dataset that was already censored for dead individuals and failed transmitters as described in [16].

We first censored the sets of records invalidated by death of individuals and transmitter failures. This entirely removed 9 individuals from set A and yielded a total of 211 individuals (168 females: 43 males) (for details see Additional file 1: Methods S1). Both data sets were then further processed and filtered before analysis. Additional file 1: Table S1 provides a breakdown of resultant sample sizes and individual numbers throughout the process.

First, successive records for each individual were combined to no more than a single movement per 24 h to prevent masking of long-range daily movements by high recording frequency. In such a case, a straight-line movement from the start point of the first movement to the end point of the last movement was assumed. This process excised 0.5% of records in data set A and 63% of records in data set B, because mean recording frequency in the latter was higher.

We then identified records that could be interpreted as the starting point of individual migration-scale movements during the autumn migration period, based on the following criteria (for details see Additional file 1: Methods S2): (1) movement distance to following recorded location ≥ 30 km, based on the approximate empirical breakpoint between local and migration flights in mallards [43]; (2) time period 1 September–31 December of each year; (3) time difference to next record ≤ 48 h; and (4) movement had a southward component.

We chose to pool the two processed data sets for further analysis, since the required location accuracy for the investigation of the migration-scale movements of interest (30+ km) was present in both sets. The pooled data set consisted of 269 relocation records representing a total of 82 individuals (48 female and 13 males from set A, 21 females from set B). The identified relocation records were used as the basis for constructing the set of alternative departure dates for discrete choice analysis.

### Discrete choice modeling

We conceptualized migration as a choice that individual animals make based on ambient environmental conditions. To model the selection of departure dates by individual mallards, we used discrete choice models [41, 44, 45]. A choice set included a date that an animal selected to migrate and a matched suite of available alternative dates the duck did not migrate. Conceptually, animals assign separate utilities *U* to each alternative date in a choice set. The alternative with the highest utility has the greatest probability that the animal will select that alternative to migrate. Although the utility of any given alternative can be negative, the above concept remains unchanged: alternative departure dates with higher utilities have an increased probability of departure compared to alternatives with lower utilities. We modeled utility as an additive linear combination of covariates, wherein *U* of alternative *j* in choice set *i* by animal *a* based on *k* = 1…*K* covariates can be written as:

$$U_{aij} = z_{aij1} b_{a1} + \cdots + z_{aijK} b_{aK} + \varepsilon_{aij} = \underline{z}_{aij}^{\prime } \underline {b}_{a} + \varepsilon_{aij}$$

with errors *ε*_*aij*_ following the Gumbel extreme value distribution. In generalized linear terminology, *U* is the linear predictor. In our discrete choice model, observed data values are *Y* = 1 for dates on which an animal migrated and *Y* = 0 for dates on which an animal did not migrate. In each choice set, only one alternative is selected. The expected relative probability that alternative *j* in choice set *i* will be selected by animal *a* is then the discrete choice model [40, 44]:

$$P\left( {Y_{aij} = 1|\underline {z}_{aij} } \right) = \int {\frac{{\exp \left( {\underline{{z_{aij}^{\prime } }} \underline {b}_{a} } \right)}}{{\mathop \sum \nolimits_{j = 1}^{7} \exp \left( {\underline {z}_{aij}^{\prime } \underline {b}_{a} } \right)}}f\left( {\underline {b}_{a} } \right){\text{d}}\underline {b}_{a} }$$

where $\mathop \sum \nolimits_{j = 1}^{7} P\left( {Y_{aij} = 1|\underline {z}_{aij} } \right) = 1.0$. *b*_*a*_ denotes individual-level coefficients that account for inter-individual variance in selection patterns and serve to relax the assumption of independence from irrelevant alternatives (IIA) [44]. Observed heterogeneity in this variance component can be accommodated by including individual-specific covariates (e.g., sex), or a common distribution can be employed. Given our data structure and in the absence of specific information, we assumed a normal distribution *f*(*b*_*a*_) with mean *ß*_*k*_ and standard deviation *s*_*k*_ [45].

Rather than the commonly used selection among spatially distinct resources such as habitat patches or food sources (e.g., 48–51), we employed a time series of successive records (dates) at the same location as the choice set, and modeled the utility of each date as a temporal alternative—that is, as the time at which a relocation could be undertaken. We constructed choice sets of *J* alternatives by selecting an identified relocation record and the *J*-1 non-relocation records directly preceding it. The observed chosen alternative (identified relocation) was thus always on the last day (day *J*). We used *J* = 7 as a set size that provided a balance between number of alternatives (*n* = 7), number of available choice sets containing at least this number of alternatives (*n* = 73), and number of individual mallards represented by these choice sets (43; 52% of total individuals) (Additional file 1: Table S1). Although each choice set consisted of exactly 7 records, the period between separate records could be longer than one calendar day (see above), resulting in choice sets longer than 7 days. Among sets, 36/73 (49%) covered a period of 7 calendar days, 68/73 (93%) covered up to 14 days, and the longest period was 35 days.

The final data set thus consisted of 43 individuals (39 female: 4 male), 73 relocation dates and 73 × 7 = 511 total alternative dates. For analysis, data were specified as panel data (i.e., possible repeated choices for decision makers) at the individual level to account for the fact that ~ 50% of mallards (21/43) were represented by two or more choice sets (1–4 sets per individual, with a mean of 2).

### Environmental data

After censoring, location records were matched with daily mean environmental parameter records from the National Oceanic and Atmospheric Administration’s (NOAA) North American Regional Reanalysis (NARR) database [49], using a nearest-neighbor approach based on their position within a grid of 32 × 32 km cells. This resolution corresponded closely to our chosen minimal range of 30 km for analyzed movements. We then matched each location record with the corresponding daily parameters of its grid cell. We selected 10 potentially informative variables based on their performance in previous population-level studies [12, 17, 18, 25]. Seven of these were used to describe short-term (daily) conditions at the location:

- Surface air temperature (*temp*; °C) was measured at 2 m above ground level.
- Depth of snow cover (*snow*; m)
- Difference in barometric pressure (*press diff*; Pa) was calculated between successive records.
- Total precipitation (*precip*; kg/m^2^) represented water in any form (rain, snow, freezing rain, or hail) amassed throughout the day.
- Cloud cover (*cloud*; %) represented the combination of low-, mid- and high-level clouds.
- Headwind and tailwind speed (*head* / *tail*; m/s). Wind speed was provided as separate meridional and zonal speed components, which were combined to yield a directional wind vector. This vector was then classified relative to the mean direction of all relocations (162°), with directions within 60° to either side (a 120° arc) classified as “tailwind” and the rest (a 240° arc) classified as “headwind”, and the associated wind speed assigned to the respective parameter while the other was set to 0.

We also computed three cumulative parameters descriptive of multi-day conditions:

- Frost days (*frost days*; d) were consecutive days of mean temperature < 0 °C, calculated for each individual based on the rounded number of sequential 24 h periods spent in one location while the condition “temperature < 0 °C” prevailed. Location was considered to change whenever a bird had moved a cumulative straight distance of ≥ 30 km over any number of records (i.e., left a circle of radius 30 km around the last location). If the interval between two records spanned multiple calendar days with both bounding records at the same location, we assumed that the bird remained at the location for the entire period. Movement to a new location or temperatures > 0 °C reset the counter to zero.
- Snow days (*snow days*; d) were consecutive days of snow depth ≥ 2.54 cm [1 inch], calculated equivalently to frost days.
- Ice cover (*ice cover*; yes/no) was a binary index recording the assumed presence of ice ≥ 1 cm thick on shallow water bodies. We based the calculation of ice growth on empirical formulae that reported thin ice growth of 1 cm per 3.3 freezing degree days (FDD Celsius) and melting of 1 cm per 1.3 thawing degree days (TDD Celsius) [50, 51]. This applies from initial ice formation, the speed of which depends on the heat capacity of the water body; because dabbling ducks typically feed in shallow water bodies, we assumed a conservative two days of freezing temperatures for initial ice formation. At a given location, tracking of ice thickness was thus triggered after two calendar days of mean temperature < 0 °C and reset to zero if thickness was estimated to drop below 1 cm.

Daily means were used for the computation of all parameters. We did not include seasonal effect, in the form of Julian date, as a covariate because the structure of our model implied that this parameter would monotonically increase within each choice set (see below).

To rescale environmental covariates for analysis, all were centered on the mean and divided by two standard deviations [52]. Model coefficients (*β*_*K*_) therefore represent the expected change in utility for an alternative departure day given an increase of two standard deviations of the independent variable.

### Candidate models and analysis

We used the environmental parameters as covariates to formulate a set of candidate models representing hypotheses about mallards’ choice of relocation day (Table 1). We included univariate models for each of the 10 covariates, and a full model containing all covariates. We also included separate models for testing the covariates quantified on a daily scale (model *daily scale*) and the covariates quantified over multiple days (model *multi-day scale*). Finally, we included a model with covariates that represented winter conditions, i.e., low temperatures and presence of snow and ice (model *winter conditions*). We also included a statistical null model that assumed each alternative had the same utility and thus that all alternatives had identical relative probabilities of selection. To assess multicollinearity, we calculated variance inflation factors (VIF) for each multivariate model; the maximum value (3.1) was well below the suggested threshold of 10 for assuming potentially problematic collinearity between predictors [53]. At the individual level, sex was available as a covariate. We ran all models both with inter-individual variance in covariates as driven by the discrete sex covariate, or as normally distributed across the range of each population-level covariate.

**Table 1:** Candidate models for discrete choice analysis

Model	Covariates	Notes
Null model	Choice set ID	Statistical null model
Temperature	Temp	Air temperature at 2 m (ºc)
Snow cover	Snow	Depth of snow cover (m)
Pressure difference	Press diff	Difference in barometric pressure to next record (Pa)
Precipitation	Precip	Accumulated water (rain, snow, freezing rain, hail) (kg/m^2^)
Cloud cover	Cloud	Total cloud cover (%)
Tailwind speed	Tail	Speed of wind with heading within ± 60º of mean relocation direction (m/s)
Headwind speed	Head	Speed of wind with heading greater/smaller than ± 60º of mean relocation direction (m/s)
Frost days	Frost days	Number of sequential days of temperature < 0ºc experienced by mallard at this location
Snow days	Snow days	Number of sequential days of snow depth ≥ 2.54 cm (1 inch) experienced by mallard at this location
Ice cover	Ice cover	Presence (y/n) of ice cover ≥ 1 cm on shallow water bodies
Full model	Temp + snow + press diff + precip + cloud + tail + head + frost days + snow days + ice cover	All covariates (*n* = 10)
Daily scale	Temp + snow + press diff + precip + cloud + tail + head	Shorter-term (daily) conditions
Multi-day scale	Frost days + snow days + ice cover	Longer-term (multi-day) conditions calculated as cumulative parameters
Winter conditions	Temp + snow + frost days + snow days + ice cover	Covariates specific to winter conditions (low temperature, snow, ice)

Resource selection probability functions (RSPFs) model the probability that an individual will select a resource unit with a given set of covariates when encountered, whereas resource selection functions (RSFs) model a dependent variable that is proportional to the RSFP. Assuming an exponential RSF, we calculated a selection index for the top model [*w*(*z*) = exp(*z*_1_*ß*_1_ + ⋯ + *z*_K_*ß*_K_)] which is proportional to the selection of a given alternative at the population level [44, 54]. To investigate the interactions between covariates at different magnitudes, we predicted both utilities and selection indices over the recorded range of one covariate while fixing the others either at their mean, or at the 25th (“low” value) or 75th percentile (“high” value) of their range for the top model.

We evaluated the predictive ability of models with leave-one-out cross-validation, fitting models to the data excluding a single choice set, and excluding each of the 73 choice sets in turn [55]. We then calculated the mean percentage of models where the observed day (day 7) of migration exhibited the highest utility within the choice set (*% correct*) across all validation sets. Although the percent correct does not constitute a goodness-of-fit statistic [40], we provide it as a useful index of predictive power. We ranked models by Bayesian Information Criterion (BIC) rather than by Akaike’s Information Criterion (AIC) in the interest of more conservative ranking [53]. The population-level parameter estimates *ß*_*K*_ and the standard deviations *s*_*K*_ were counted as parameters for the purpose of BIC calculation. Inferences on relocation day choice by mallards were made from the top-ranked model. We fit discrete choice models with package *gmnl* [45] in R v4.0.3 [56]. We used 500 draws of simulated probabilities in the maximum likelihood estimation, after having determined that parameter estimates remained static above this number (at the level of three significant digits).

## Results

Models with air temperature and snow conditions were highly ranked, while models conditional on wind speed, precipitation, presence of ice, pressure difference, and cloud cover received much lower support (Table 2). Model selection among the set of candidate models indicated a single top model, *winter conditions*, with a relative BIC model weight of 1.0. This model also shared the highest predictive power (60% correct over 73-fold cross-validation), together with the lower-ranked *full model*. The *null model*, representing equal selection probability for each of the seven alternatives in a set, had the expected predictive power of 14% (1/7). The highest-ranked and most predictive single-parameter model was *temperature* (ΔBIC 21.2, 52% correct). *Headwind speed* and *ice cover* had the lowest predictive power (7% and 4% respectively). We found no support for sex as a predictor of inter-individual preference, as the resultant model set showed the same rank order but globally raised BIC values (data not shown) compared to the set assuming a normal distribution of preferences across individuals. All inferences are therefore based on the highest-ranked model (*winter conditions*) of the latter set.

**Table 2:** Model selection table

Model	*K*	% Correct	BIC	ΔBIC
Winter conditions	10	60	223.3	0
Multi-day scale	6	42	242.8	19.5
Temperature	2	52	244.5	21.2
Snow days	2	18	246.6	23.3
Snow cover	2	30	248.6	25.3
Full model	20	60	251.3	28
Frost days	2	40	252.5	29.2
Tailwind speed	2	36	257.1	33.8
Daily scale	14	53	263.2	39.9
Precipitation	2	27	275.7	52.4
Ice cover	2	04	277.9	54.6
Pressure difference	2	32	279.2	55.9
Headwind speed	2	7	279.6	56.3
Cloud cover	2	19	281.1	57.8
Null model	2	14	292.7	69.4

Models are ranked by increasing BIC. *K*, number of parameters used for BIC calculation; % correct, proportion of observed relocation days (day 7) correctly predicted with leave-one-out cross-validation; ΔBIC, difference in BIC value to top model

**Fig. 1:**
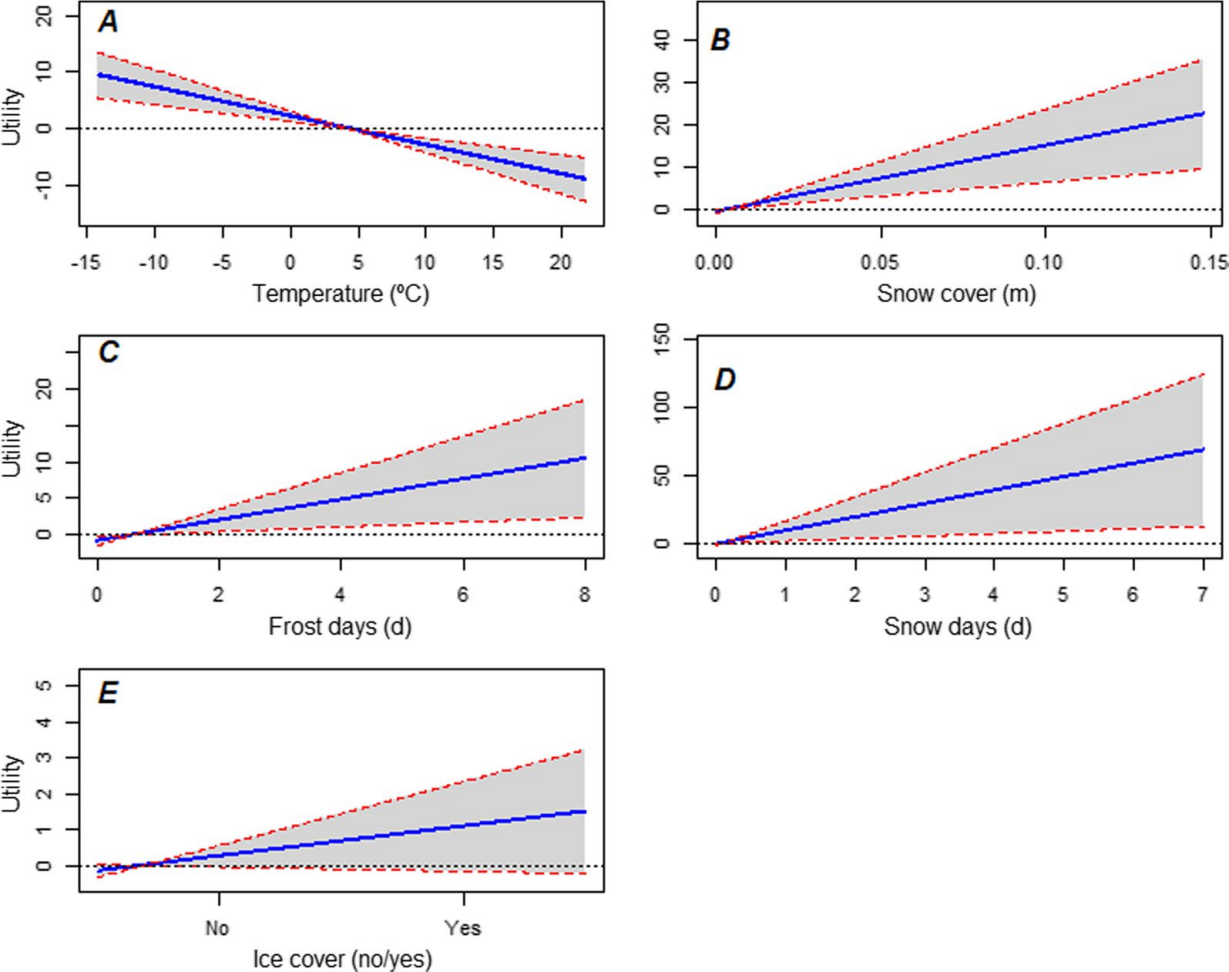
Mean predicted utility ± 1 standard error (shaded area) for a resource unit as a function of covariate value over the observed range, based on the top model. All other covariates were held at mean values to calculate predictions. **A** Air temperature; **B** depth of snow cover; **C** accumulated days of frost; **D** accumulated days of snow ≥ 2.54 cm deep; **E** presence of ice cover ≥ 1 cm thick. Note different y-axis ranges. Negative slope values represent a *decrease* in the probability that the associated alternative is selected as the date for relocation

**Fig. 2:**
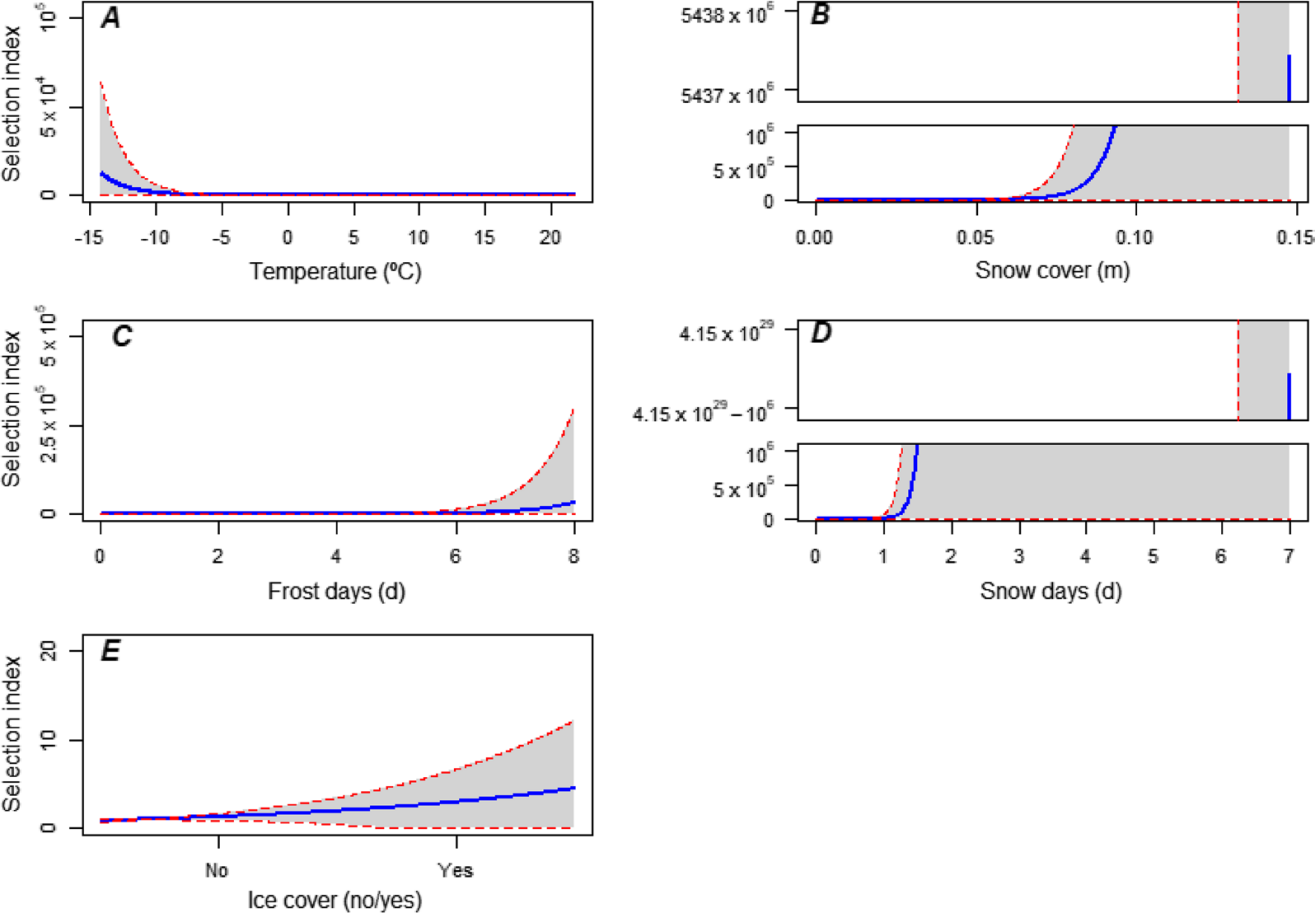
Mean predicted selection index ± 1 standard error (shaded area) as a function of covariate values in the top model (*winter conditions*). All other covariates were held at mean values to calculate predictions. The selection index is proportional to an alternative’s selection probability given this set of covariate values. **A** air temperature; **B** depth of snow cover; **C** accumulated days of frost; **D** accumulated days of snow ≥ 2.54 cm deep; **E** presence of ice cover ≥ 1 cm thick. Note different y-axis ranges

**Fig. 3:**
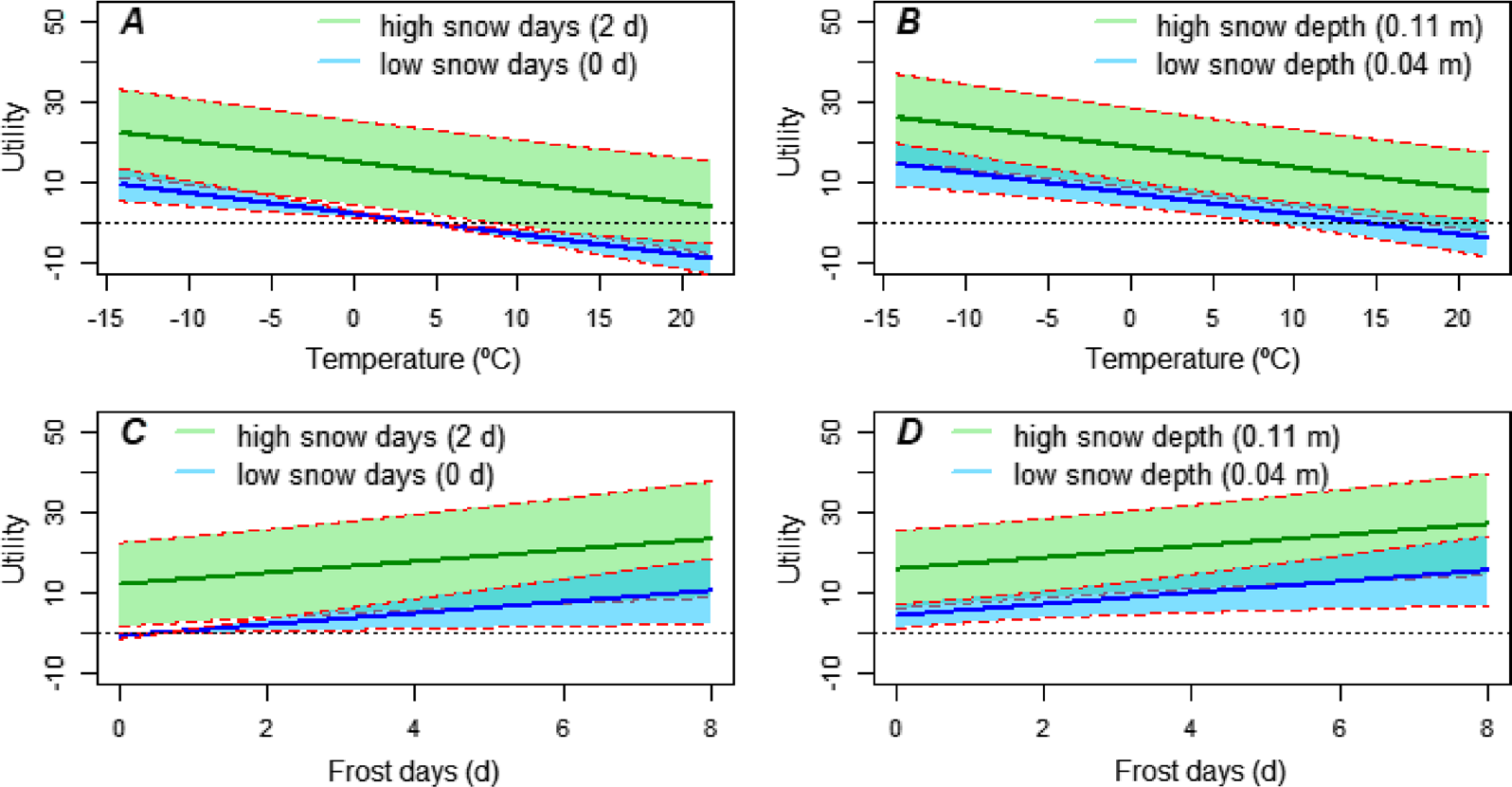
Mean predicted utility ± 1 standard error (shaded area) for a resource unit in the top model (*winter conditions*) as a function of *temperature* or *frost days*. **A** utility as a function of temperature, given high (green) or low (blue) values of snow days; **B** utility as a function of temperature, given high (green) or low (blue) values of snow cover depth. **C**, **D** equivalent for utility as a function of *frost days*. All other covariates were held at mean values to calculate predictions

**Fig. 4:**
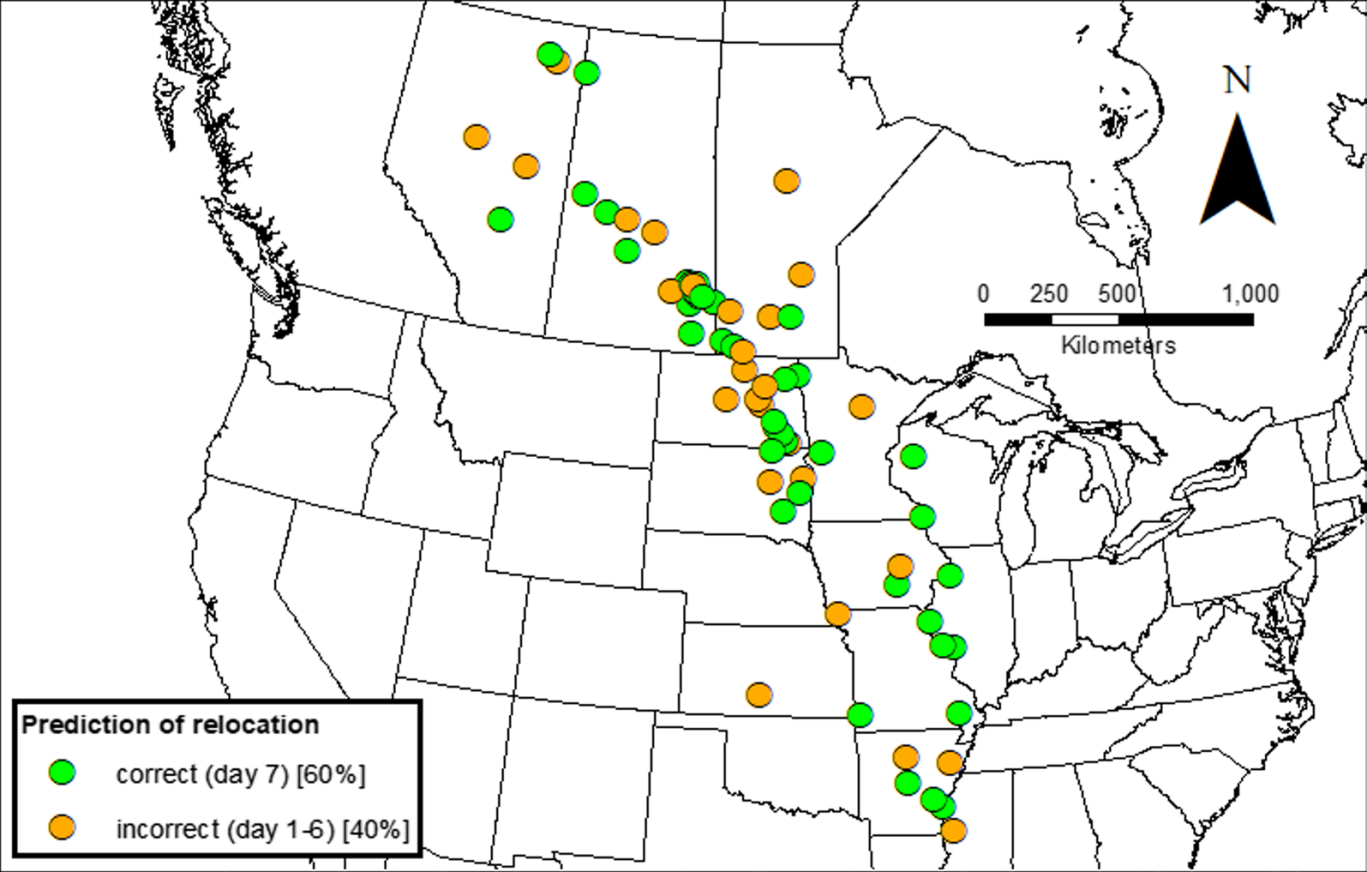
Location of start points of observed mallard relocations (*n* = 73), indicating whether the day of relocation (day 7) was correctly predicted from the relevant choice set

The winter conditions model included temperature, depth of snow cover, number of frost days experienced, number of snow days experienced, and presence of ice cover (≥ 1 cm thick) (Table 1). Specifically, temperature exhibited a negative relationship with the probability of departure ($\hat{\beta }$ =  − 6.04, SE = 2.59), indicating lower temperatures increased the probability of a duck choosing to migrate (Figs. 1A, 2A). In contrast, snow cover ($\hat{\beta }$ = 5.23, SE = 3.04) and frost days ($\hat{\beta }$ = 3.71, SE = 2.86) exhibited positive relationships with the probability of departure, indicating that greater snow cover and number of frost days increased the probability of migrating (Figs. 1B, C, 2B, C). The presence of ice cover ≥ 1 cm thick had the smallest positive relationship with probability of departure ($\hat{\beta }$ = 1.65, SE = 1.90) (Figs. 1E, 2E). The number of snow days had the largest effect on probability of departure ($\hat{\beta }$ = 10.61, SE = 8.68) with additional snow days generating a greater probability of migrating, but also exhibited high uncertainty (Figs. 1D, 2D); this parameter also had the greatest VIF (2.6) in the model. Substantial snow (greater numbers of snow days or greater snow depth) dominated choice of migration date to the extent that temperature parameters (frost days and temperature) had minimal impact, whereas under low snow conditions, migration probability depended on temperature conditions (Fig. 3). Inter-individual variability in preference among mallards was pronounced for temperature (*ŝ* = 9.34, SE_*ŝ*_ = 3.41) and frost days (*ŝ* = 8.20, SE_*ŝ*_ = 3.13), lower for depth of snow cover (*ŝ* = 5.71, SE_*ŝ*_ = 5.63) and snow days (*ŝ* = 2.42, SE_*ŝ*_ = 5.38), and almost absent for ice cover (*ŝ* = 0.33, SE_*ŝ*_ = 3.03) (Additional file 1: Fig. S1). Residuals of the top model (median = 0.045, SD = 0.275) fit a Gumbel extreme values distribution poorly (Kolomogorov-Smirnov test, *p* < 0.001). However, residuals were small relative to utility estimates (median = 0.152, SD = 5.344), indicating that the assumption was not overly restrictive [40]. There was no discernable difference between the distribution of all relocation latitudes (*n* = 73) and the distribution of latitudes where relocation was predicted correctly (Fisher-Pitman permutation test, 10^5^ samples; *n* = 45, *Z* = 0.129, *p* = 0.898) or incorrectly (*n* = 28, *Z* =  − 0.173, *p* = 0.863) (Fig. 4).

## Discussion

We investigated the decision of individual mallards during autumn migration in the Mississippi Flyway to relocate south, as a function of environmental covariates that were descriptive of foraging habitat conditions and flight weather. Our results indicated that relocation probability was primarily influenced by winter conditions characterized by snow cover in the previous few days and current snow cover depth. Mallards reacted most strongly to experienced snow days, with even a single snow day resulting in very high relative relocation probability (Fig. 2D). Responses to snow cover depths above 7 cm were similarly pronounced (Fig. 2B). At low snow cover or in absence of snow days in the recent past, temperatures dropping below 5 °C were an increasing incentive to relocate; whereas under snow conditions, relocation became likely at any temperature (Fig. 3A, B). Similar dynamics were predicted for experienced frost days (Fig. 3C, D). Among evaluated variables, the effect of temperature had the smallest uncertainty.

These results indicate a ranking of responses that has been suggested by several recent studies [12, 18, 57, 58]. Decreasing temperatures and the onset of frost may act as an early warning sign that elicits a response before foraging is actively impacted. Schummer et al. [12] developed a weather severity index composed of parameters similar to those in our top model (location-based cumulative snow and frost days, and current temperature and snow cover), and found that dabbling duck abundance at Missouri sites was principally correlated to the daily temperature component. Similarly, Xu and Si [18] found that greater white-fronted and swan geese timed their southwards departure from Northern Asian stopover sites by the onset of freezing temperatures, generally leaving before snow conditions became a factor. This more pronounced reaction to frost than to snow conditions was not present in our model results; however, food availability for mallards is directly diminished by snow cover on the ground in certain regions [59], and when encountered may force birds to relocate rapidly to avoid the loss of foraging opportunities [2].

Models yielded relatively large error estimates for the snow parameters (snow days and snow cover depth) due to the strongly right-skewed distribution of these data, with 99.5% of records showing values of respectively 0–3 days and 0–10 cm, and only a few records of up to 7 days and 15 cm (data not shown). While this resulted in substantial uncertainty in prediction at higher values (Figs. 1, 2), it has little impact on interpretation due to the rarity of these instances and mallards’ strong response to lower-value snow metrics (Fig. 2B, D). Allowing for this caveat, our results are consistent with a strong and rapid reaction to snow cover, and a more graduated response to temperature cues. Mallards may have a more pronounced tendency than other dabbling ducks to remain on location in the face of worsening conditions, and sometimes leave only in the event of snowfall [12, 60]. In addition to having a comparatively high body weight, mallards can also take advantage of non-wetland food sources like harvested fields [6] and are thus less dependent on ice-free shallow water than wetland-obligate dabbling ducks. This may partly explain why presence of ice cover, included in the top model as a putative “winter conditions” parameter, was found to have no notable effect on selection of relocation date (Figs. 1E, 2E); another likely cause was the low accuracy of the heuristics we used to calculate this metric, and the absence of a method to estimate fractional ice coverage of water bodies.

We found greater inter-individual variance in selection for temperature-related metrics (temperature, *ŝ* = 9.34; frost days, *ŝ* = 8.20) than for snow-related metrics (snow cover, *ŝ* = 5.71; snow days, *ŝ* = 2.42), possibly reflecting differences in condition ranges tolerated by individual mallards during the evaluated time periods close to relocation (Additional file 1: Fig. S1). Thus individual mallards might tolerate a variety of above- or below-freezing temperatures and none to several frost days before relocating, but rarely more than two snow days or a light snow cover. Differences in body mass or condition may play a role in this regard. Previous studies have shown that female mallards tended to remain at stopover sites longer [14] and migrated farther and arrived later than males [61], indicating a possibly greater tolerance for worsening weather conditions. However, we found no correlation between individual preferences and sex in our model, presumably because the data set was heavily skewed towards females (39:4), making it unlikely that sex differences could be reliably distinguished.

We hypothesized that mallards’ functional responses to environmental parameters would apply at any point during migration and in the wintering range, and therefore included relocation instances from across the whole modeled migration period (September-December) in our model. We found no discernable latitudinal differences between the set of all relocations and those of correctly or incorrectly predicted relocations (Fig. 4). This suggests that the identified responses to environmental conditions hold equally for departure from the summer range, stopover sites, and early relocations within the winter range. Similarly, van den Elsen [58] and Schummer et al. [10] found that the Schummer et al. [12] weather severity index was applicable throughout the latitudinal range of several duck species including mallards, although prediction could be further improved by adding a latitude parameter to the model [58].

Across the set of candidate models, the temperature and snow covariates, which could be considered descriptive of habitat availability, performed much better than the flight weather covariates that would be expected to influence short-term departure decisions (Table 2). In contrast, Xu and Si [18], who included snow and frost days and vegetation indices together with wind and precipitation in their goose migration model, concluded that tailwind conditions played a substantial role in predicting departure from stopover sites. O’Neal et al. [17] examined the influence of numerous flight weather parameters on the departure probability of a diverse group of dabbling ducks from an Illinois site, and found that the dominant predictors consisted of tailwind, absence of precipitation, and low cloud cover; however, the only multi-day or habitat-related parameter tested was a vegetation index. The departure-promoting effects of tailwinds in particular are well established for several goose species [29, 30, 62, 63], if less so for ducks. It is likely that in dabbling ducks, as in other bird species, large-scale environmental cues like habitat conditions drive willingness to depart at a daily or larger scale, while flight weather influences timing at a daily or smaller scale [2, 25, 64]. Our data were not well suited to model this distinction because the dynamic positioning of mallard departure locations (rather than recording at fixed sites) required the use of environmental data at a relatively coarse resolution (32 × 32 km, daily averages), which likely affected flight weather data more than multi-day habitat-related metrics and favored the impact of the latter. It is notable that while the candidate model bundling the short-term parameters (*daily scale*) was ranked well below the multi-day parameter models, it still achieved 53% predictive power (Table 1). We did not model interactions between the two classes of parameters because of our relatively low sample size (73 choice sets); investigating these relationships using a larger individual-based data set might be of considerable interest.

A benefit of data derived from individual tracked birds is the availability of cumulative parameters in each individual’s frame of reference rather than that of a visited location, which may be more relevant to the decision-making process. We thus calculated sequential snow and frost days as experienced by each mallard from the time of arriving at a location, in contrast to the location-continuous values used by Schummer et al. (2010, 2017) [10, 12]; however, differences between these two types of metrics were minimal in our choice sets (present in 3/73 sets; not shown) because the requirement for mallards to remain on location for multiple days prior to departure selected against locations that were already under winter conditions on arrival.

Snow as a principal migration driver is likely to be affected by warming global temperatures in the coming decades. In the Midwest region of North America, regional annual projections for the mid-twenty-first century average an increase of 2.3–2.9 °C [65], with winter temperatures from December to March increasing by 1.1–3.9 °C [66]; various regional models project reduced snowfall that may lower the number of days with snow cover of at least 1 cm by between 5 and 60 days annually [67]. The majority of investigations into the effects of climate change on duck migration have focused on spring migration, where warm conditions earlier in the year may allow better body condition and earlier arrival in the breeding range, but may also lead to a phenological mismatch between breeding stages and peak food availability [9]. However, the weather during autumn migration and the mid-winter period is also likely to be affected by climatic changes. Recent northwards shifts in the wintering distribution of diverse waterfowl and wader species have been documented (e.g. [68, 69]). Schummer et al. [10] found that the extent of areas in the Mississippi and Atlantic flyways that had winter conditions severe enough to cause mallards to relocate in the period December-February had declined from 1979 to 2013 [10]. Sauter et al. [60] reported that mid-winter movement distances of European mallards decreased between 1952 and 2004, likely due to less frequent occurrence of harsh winter conditions [60]. In North America, some studies have reported evidence that the winter distributions of North American mallards are shifting northwards [11, 70] (although these findings are based on less accurate convenience-sampled data; see also [71]). If birds remain longer at more northern latitudes in autumn and winter, this may increase foraging pressure in northerly areas and require adaptation in regional conservation planning to meet changing nutritional needs at the landscape level [72].

A substantial percentage of relocation choices remained unexplained by modeled parameters. The chosen temporal and spatial scales may have masked smaller-scale variations in weather and environmental conditions. Although our study was the first to examine and conceptualize waterfowl migration as an individual choice, the dataset and model structure limited our ability to include choice set-level parameters such as measures of wetland flooding status or vegetation cover, which may strongly affect habitat suitability for foraging [43]. The presence or absence of conspecifics may have influenced departure decisions through competition and food depletion, or by participation in flock movements. Disturbance by hunters also has been shown to factor into the timing of long-range relocation movements [73, 74]. Finally, we had no information of the body condition of individuals, which may have influenced length of stay at productive foraging sites, willingness to embark in energetically costly flight weather, and flight duration. The collection of metabolic data from free-living birds, while still complicated and costly, is becoming more feasible with the ongoing miniaturization of implantable bio-loggers [75]. Inferences about body condition and energy budget derived from such data can provide valuable additional information for the interpretation of movement records from tracked animals [76, 77]. Future research into North American waterfowl migration focusing on combining high-resolution location records with disturbance and metabolic data would be helpful for developing a comprehensive picture of the drivers of migratory decisions.

## Conclusions

Our results show that among the tested environmental parameters influencing departure decision in autumn-migrating mallards, the dominant driver was the onset of snow conditions, and secondarily the onset of temperatures lowering close to or below the freezing point. Mallards are likely to relocate southwards quickly when faced with foraging impeded by snow, and use declining temperatures as a more graduated early cue for departure. The effects of short-term weather conditions presumed to be related with flight efficiency could not be distinguished in the model. Our findings provide further insights into the response of mallards to weather and climate factors during the migration period, and will be useful in the prediction and simulation of dabbling duck migratory movements under changing climatic conditions.

## Supplementary Information

**Additional file 1**. Supplementary Methods (censoring of positional data sets, and relocation record selection criteria) and Results (inter-individual selection variance in the top model).

## Data Availability

The datasets used and/or analysed during the current study are available from the corresponding author on reasonable request.
