# Supplementary material for: Environmental drivers of autumn migration departure decisions in midcontinental mallards
Source: Mov Ecol. 2022 Jan 5;10:1. doi: 10.1186/s40462-021-00299-x (PMC8729067; doi:10.1186/s40462-021-00299-x)
Supplement: Supplementary file 1 — Additional file 1. Supplementary Methods (censoring of positional data sets, and relocation record selection criteria) and Results (inter-individual selection variance in the top model). [file 40462_2021_299_MOESM1_ESM.docx]

**Additional file 1**

**Additional file: Methods**

**S1 - Censoring of data sets for death of individuals and transmitter failure**

Record time series for individual birds were terminated when it was deemed probable that the individual had died, or that the transmitter had ceased to accurately transmit location. This determination was based on two criteria: a) absence of movement for more than two consecutive records of the transmitter’s on-board activity counter; and b) absence of movement for more than five consecutive calendar days as determined by geospatial coordinates. Records were categorized by the monitoring system into classes of estimated location accuracy. We excluded only records of class Z (no error estimates available and flagged as unreliable) and retained other records of classes that have been shown to yield mean location error estimates well within the 30 km movement range of interest (classes 3, 2, 1, 0, A, and B) by Costa et al. (2010) (1). In that study, the empirical error of each location class (LC) was tested in the field by comparison against Fastloc GPS records from the same electronic tags on a range of pinniped species. The 68^th^ percentile ARGOS location errors estimated by the authors were as follows: LC-3: 0.49 km; LC-2: 1.01 km; LC-1: 1.20 km; LC-0: 4.18 km; LC-A: 6,19 km; LC-B: 10.28 km.

We found no substantial correlation of location class with either distance or time period between sequential records, and therefore did not further distinguish between classes in the analysis.

**S2 - Criteria for selection of autumn migration relocation records**

The following criteria were used to identify individual location records representing the start points of migration-scale relocation movements during autumn migration.

1. *Movement distance to following record ≥ 30 km*. This is based on the finding of Beatty et al. (2014) (2) that this distance represents the approximate breakpoint between local foraging flights and relocation flights in mallards, independent of time of year. Straight line distances were calculated as great circle distances assuming a spherical geoid.
2. *Time period 1 September – 31 December*. We excluded records prior to the period during which autumn migration movements would be expected. While we specifically included December records to capture relocation movements in response to environmental changes in the wintering range, we set a cut-off at 31 December to avoid conflation with early spring migration activity.
3. *Time difference to next record ≤ 48 h.* Many records in the censored and concatenated sets had recording intervals in excess of 24 h (82% in set A, 6% in set B). For long-range relocations spanning a large time interval, the uncertainty in assigning environmental parameters to the start record would increase with the interval length, because it could not be determined at which point the mallard actually embarked on the relocation (e.g. for a ten day interval T_1_ → T_10_, whether relocation actually happened on T_1_, or on T_2_,…,_9_). We therefore excluded relocations with intervals in excess of 48 h. For the remainder, daily environmental parameters assigned to the start date were averaged across the first two days of the interval if the interval spanned three rather than two calendar days.
4. *Movements with southward component.* Finally, we excluded relocations where the movement vector did not have any southward component (i.e. did not include any decrease in latitude), to ensure that we only captured relocations connected to autumn migration behavior.

Additional file 1: Table S1. Censoring and filtering of mallard location record sets

|  | **Data set A** | **Data set B** |
| --- | --- | --- |
| number of records after restricting to: |  |  |
| - live individuals / active transmissions | 5603 | 8271 |
| - no more than one movement per 24 h per individual | 5573 | 3026 |
| number of individual mallards   - in original data set | 180  (134 f : 46 m) | 40  (female) |
| - in restricted data set | 171  (128 f : 43 m) |  |
|  | | |
|  | **Data set A - relocations** | **Data set B - relocations** |
| number of records after restricting by autumn migration relocation criteria: |  |  |
| - movement ≥ 30 km | 737 | 214 |
| - annual period 1 Sep – 31 Dec | 592 | 151 |
| - time difference between records ≤ 48 h | 265 | 111 |
| - movement with southward component | 173 | 96 |
| total number of relocations | 269 | |
| number of individual mallards represented by relocations | 61  (48 f : 13 m) | 21  (female) |
|  | 82 [pooled] | |
|  | | |
|  | **Choice set data** | |
| choice sets with ≥ 7 alternatives (i.e., relocations with ≥ 6 preceding records at location) | 73 | |
| total number of alternatives | 511 | |
| number of individual mallards represented by choice sets | 43 (39 f : 4 m) | |
| years covered | 2004, 2005, 2006, 2007, 2010, 2011 | |
| median relocation distance on day 7 | 153.6 km | |

**Additional file: Results**

Additional file 1: Fig S1. Inter-individual variance in preference for environmental covariates in the top model (*winter conditions*). x-axis: mean parameter estimate; y-axis: kernel density across individuals. Grey shaded area indicates proportion of individuals with a positive mean utility for this covariate.


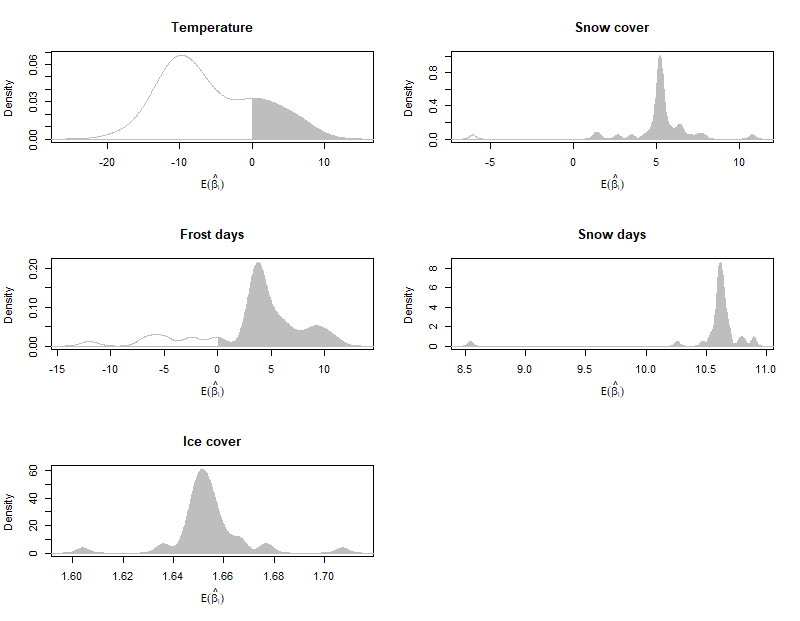


**Additional file: References**

1. Costa DP, Robinson PW, Arnould JPY, Harrison A-L, Simmons SE, Hassrick JL, et al. Accuracy of ARGOS locations of pinnipeds at-sea estimated using Fastloc GPS. Ropert-Coudert Y, editor. PLoS ONE. 2010 Jan 15;5(1):e8677.

2. Beatty WS, Webb EB, Kesler DC, Raedeke AH, Naylor LW, Humburg DD. Landscape effects on mallard habitat selection at multiple spatial scales during the non-breeding period. Landscape Ecol. 2014 Jul;29(6):989–1000.
